# Association between Psychological, Biochemical and Personal Factors with the Inflammatory Marker High-Sensitive C Reactive Protein (Hs-CRP) in Mexican Healthy Population

**DOI:** 10.3390/jpm13050876

**Published:** 2023-05-22

**Authors:** Aniel Jessica Leticia Brambila-Tapia, Ana Lilia Jacquez-Castañeda, Laura Arely Carrillo-Delgadillo, Jessica Natalia Dávila-Flores, Fabiola Macías-Espinoza, Saúl Ramírez-De Los Santos, Itzae Adonai Gutiérrez-Hurtado

**Affiliations:** 1 Departamento de Psicología Básica, Centro Universitario de Ciencias de la Salud (CUCS), Universidad de Guadalajara, Guadalajara 44340, Jalisco, Mexico; 2 Maestría en Psicología de la Salud, Centro Universitario de Ciencias de la Salud (CUCS), Universidad de Guadalajara, Guadalajara 44340, Jalisco, Mexico; lilia.jacquez2247@alumnos.udg.mx; 3 Licenciatura en Psicología, Centro Universitario de Ciencias de la Salud (CUCS), Universidad de Guadalajara, Guadalajara 44340, Jalisco, Mexico; 4 Licenciatura en Nutrición, Centro Universitario de Ciencias de la Salud (CUCS), Universidad de Guadalajara, Guadalajara 44340, Jalisco, Mexico; 5 Departamento de Psicología Aplicada, Centro Universitario de Ciencias de la Salud (CUCS), Universidad de Guadalajara, Guadalajara 44340, Jalisco, Mexico; 6 Departamento de Biología Molecular y Genómica, Centro Universitario de Ciencias de la Salud (CUCS), Universidad de Guadalajara, Guadalajara 44340, Jalisco, Mexico

**Keywords:** hs-CRP, anxiety, depression, neuroinflammation, inflammation, psychological factors, biochemical variables

## Abstract

In the last decades, it has been shown that inflammatory processes play a role in the development of mental and physical problems; although some studies have researched the relationship between inflammation and psychological variables, the inclusion of biochemical variables as possible confounders has been limited. Therefore, the aim of this study was to determine whether psychological variables are associated with the inflammatory marker, highly sensitive CRP (hs-CRP), after controlling for personal and biochemical variables in the Mexican population. The study was performed at the University of Guadalajara facilities, during the second half of 2022. Healthy subjects were invited to participate in the study, which consisted of the measurement of personal, psychological, and biochemical variables. We included 172 participants, 92 (52.9%) of which were women; the median (range) of age of the whole sample was 22 (18–69) years old. In the bivariate analysis, significant positive correlations were observed between hs-CRP and body mass index (BMI) and waist/hip ratio (WHR) in both sexes, together with leukocytes, uric acid, low-density lipoprotein (LDL), triglycerides, and the liver enzymes gamma glutamyl transferase (GGT) and alkaline phosphatase (ALP). In the multivariate regression analysis of the global and men’s samples, anxiety was positively associated with hs-CRP, while depression and positive relations with others were negatively associated with hs-CRP. In conclusion, psychological variables influence inflammation mainly in men, and anxiety was the main contributor; in addition, positive relation with others is a variable that should be further explored as a psychological protector of inflammation in both sexes.

## 1. Introduction

It has been shown, during the last decades, that the immune system and inflammatory processes play a role in the development of mental and physical problems, with an important impact in morbi-mortality worldwide, with chronic diseases being the most significant cause of death around the world [1]. In addition, the presence of systemic chronic inflammation has been associated with oxidative stress, and both have been proposed as important causes of most chronic conditions, including diabetes, cancer, cardiovascular and neurodegenerative diseases [2]. Therefore, systemic chronic inflammation, together with oxidative stress, have become important research targets for preventive and therapeutic programs [1,2].

Focusing on the relationship between inflammation and psychological variables, we found that many reports have studied the association of genetic, environmental, psychological, and personal factors with inflammatory markers [3,4,5,6,7,8]. Among the main inflammatory markers studied are the C reactive protein (CRP), inflammatory interleukins IL-1 and IL-6, and tumor necrosis factor alpha (TNF-α) [6,9]. CRP is an inflammatory protein synthetized by Kupffer cells in the liver, in response to acute inflammatory events, and depending on their values, it can be indicative of chronic inflammatory states [10,11,12]. This biomarker, which has the advantage to be easily measurable in plasma, has been one of the most studied markers in relation to psychological variables, where some authors have found association between anxiety and depression levels (considered negative psychological variables) with higher levels of CRP [6,7,13], while other studies have found a negative correlation between CRP and positive affect and other positive psychological variables, including life satisfaction and psychological wellbeing, after adjusting for confounders [3,4,5]. These studies have been performed in healthy and/or ill populations and with very large sample sizes; in addition, most of them have included the main confounding variables, such as age, sex, body mass index (BMI), smoking and alcohol consumption, some of which have been associated with CRP (mainly BMI). Furthermore, many of these studies have performed comparisons separated by sex, obtaining more specific results [8,14].

However, although some studies have included the measurement of biochemical variables as possible confounders in the determination of the association between psychological factors and CRP [15,16], no studies were found (in any population) that included a wide range of these variables as possible confounders in order to better determine the real association between psychological variables and CRP. In this sense, the inclusion of biochemical variables, such as the number of leukocytes, uric acid, liver, and kidney function parameters, as well as the presence of erythrocytes in urine (present in menstruation), among others, would give a better approach to determine the main associated variables with the inflammatory marker CRP in general and in each sex; while considering that some of these parameters are related with infections or inflammatory conditions that would increase CRP. This analysis could permit to corroborate or discard the previous associations reported between studied psychological variables, including positive affect, depression and anxiety [5,6,8] and CRP. Therefore, the aims of this study were to determine whether psychological variables are associated with CRP after controlling for personal and biochemical variables in a sample of relatively healthy Mexican population. Our hypotheses are therefore: (a) after controlling for confounders (personal, anthropometric and biochemical variables), anxiety and depressive symptoms are positively correlated with CRP, and positive affect is negatively correlated with it, and (b) other positive psychological variables, not previously explored (including subscales of psychological wellbeing, emotional intelligence and optimism), are negatively correlated with CRP. These analyses are expected to be performed in the global sample and separated by sex, in order to detect any sex differences in the possibly observed associations.

## 2. Subjects and Methods

### 2.1. Subjects

The inclusion criteria of the study were: (a) subjects older than 18 years old, (b) without chronic or acute diseases (known by the subject), (c) who were not consuming illegal drugs (including marijuana), (d) who were not consuming hormonal products to increase muscular mass, (e) who were not pregnant, (f) who were not genetically related with another participant of the study (i.e., brothers, cousins), and (g) who preferably did not smoke. These requirements were used in order to diminish confounding factors. The elimination criteria were the absences of the measurements of any variable.

### 2.2. Study Design: Cross-Sectional Study

#### 2.2.1. Procedures

The study was conducted from August to November of 2022. A detailed explanation of the procedures was given to all the subjects invited. The invitation was performed via social networks (WhatsApp, Facebook and personally to the University students). All the subjects that met the inclusion criteria and accepted to participate were cited (in groups from 11–20 participants) in the facilities of the University of Guadalajara, where they signed an informed consent and filled an electronic questionnaire that included personal and psychological variables; these measurements were performed in a computer room inside the University. After the filling of that questionnaire, the height and weight were obtained by trained students with a Tanita brand scale (model bc-533) and a measuring tape stuck to the wall, in order to calculate the BMI. The hips and waist circumferences were also obtained by trained students, using a measuring tape, in order to calculate the waist/hip ratio (WHR). The blood and urine samples (to perform the laboratory tests) were obtained by qualified personnel (three biochemicals) who worked for a certified laboratory; they were attending to one participant at a time. After obtaining the samples, these were transported to a certified biochemical laboratory, where the biochemical analyses were performed by trained personnel.

**Figure 1:**
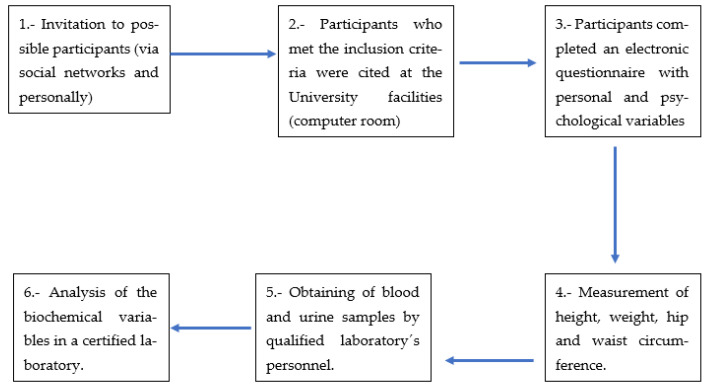
Flowchart of the procedures performed to the participants.

Around 7 students were helping with the questionnaire filling and anthropometric measurements. All the procedure lasted around one hour for each participant. The flowchart of the procedure is exemplified in Figure 1.

#### 2.2.2. Sample Size

The sample size was calculated with the formula for correlations described by Díaz-Pértegas [17], which yielded a total of 47 subjects, in order to detect as significant a moderate correlation of 0.4. However, the minimum sample size intended was 80 individuals per sex.

### 2.3. Ethical Considerations

The study was conducted according to the guidelines of the Declaration of Helsinki and was approved by the ethical committee of the Health Sciences University Center, with the registration number 19–21. All the participants signed an informed consent.

### 2.4. Personal Variables

The personal and sociodemographic variables measured were age, sex, schooling, whether they had a romantic partner, whether they had a job, socioeconomic level, daily free hours, daily hours of physical activity, monthly extra money (5 categories, from nothing to more than 150 dollars), and alcohol and smoking consumption frequency (5 categories, from never to 4 or more times in the week). Sleep satisfaction was measured with the first item of the OVIEDO sleep questionnaire, from 1 (very unsatisfied) to 7 (very satisfied); sleep quality was measured with the second item (consisting of 5 items) of the OVIEDO sleep questionnaire, from 1–5 (low quality to high quality) [18]. The quality of food intake was measured with the Mini-ECCA scale, from 1–12 (very low quality to very high quality) [19]. Additionally, as previously mentioned, the anthropometric variables BMI and WHR were obtained.

### 2.5. Psychological Variables

The following psychological variables were measured: depression, with the 10-item CES-D scale, from 1–4 (none day to all the days) [20,21]; anxiety with the Generalized Anxiety Disorder test (GAD-7), from 0–3 (never to almost all the days); ref. [22]; positive and negative emotions with the positivity-self scale (PSS), from 1–5 (never to almost always) [23]; the 6 subscales of the shortened version of the psychological well-being (PWB) scale (self-acceptance, autonomy, environmental mastery, personal growth, positive relations with others and purpose in life), measured from 1–6 (totally disagree to totally agree); ref. [24]; and optimism with the Life Orientation Test (LOT-R), from 1–5 (totally disagree to totally agree) [25]. We measured 5–6 items of 4 subscales of the Trait Emotional Intelligence Questionnaire (TEIQUE): self-motivation (5 items), emotion perception (5 items), assertiveness (6 items) and emotion regulation (6 items), from 1–7 (totally disagree to totally agree); these items are described in Supplementary File S1 [26].

### 2.6. Laboratory Tests

The laboratory tests measured, most of which included biochemical variables, were: (1) a complete blood count test (including hemoglobin, platelets, leukocytes and their subpopulations: lymphocytes, neutrophils, monocytes, eosinophils, basophils), whose measurements were performed with impedance and cytometry methods; (2) a complete lipid profile test, including total cholesterol, low-density lipoprotein (LDL), high-density lipoprotein (HDL), and triglycerides; (3) a liver function test, including aspartate aminotransferase (AST), alanine aminotransferase (ALT), gamma glutamyl transferase (GGT), alkaline phosphatase (ALP), lactate dehydrogenase (LDH) enzymes and direct and indirect bilirubin; (4) blood chemistry, including glucose, creatinine, urea, blood urea nitrogen (BUN) and uric acid; and (5) a general urine test, including leukocyte esterase, proteins, nitrites, erythrocytes and leukocytes, whose measurements were performed with colorimetry and microscopy methods. The high-sensitivity CRP (hs-CRP) was also measured with the ELISA technique. These analyses were performed in a private and certificated laboratory (with EMA and Joint commission international certifications), located in the city of Guadalajara.

### 2.7. Statistical Analysis

In order to describe the numerical variables, we used mean and standard deviations when the distribution was parametric and median and ranges for some variables with non-parametric distributions, depending on the variable (on how the variable was better described). In order to detect the distribution of the variable, we used the Kolmogorov–Smirnov test. To compare sociodemographic variables between sexes, we used the chi-squared test for qualitative variables and the Student *t*-test or Mann–Whitney U test for quantitative ones. In order to compare quantitative variables with hs-CRP, we used the Spearman correlation test, considering the non-parametric distribution of hs-CRP. In addition, multiple linear regression analysis (using the stepwise method) with the hs-CRP as the dependent variable was performed for all the sample and segmented by sex.

For this analysis, we obtained the R and R^2^ of the model, as well as the change in R^2^ observed for each variable included in the model. This change in R^2^ refers to the contribution to the variability of hs-CRP that each included variable adds to the final model. In addition, we obtained the tolerance values for each variable included in the model. In this case, tolerance values > 0.3 were considered acceptable because it indicates that there is not interrelation (collinearity) between the variables included in the model, assuming in this way, that each contributing variable in the model is independent from each other.

Finally, the Cronbach alpha test was obtained for all the psychological instruments (including the subscales) in order to obtain the reliability of each scale applied. All analyses were performed with the software SPSS v.25, and a *p* value < 0.05 was considered as significant.

## 3. Results

A total of 172 participants were screened and no one was excluded; of these, 91 (52.91%) were women and the median and range of the age was 22 (18–69) years old. The descriptive data of sociodemographic and psychological variables are described in Table 1 and those of biochemical variables, including hs-CRP, are presented in Table 2. We detected many cases with biochemical variables out of the normal range, with 18% of the sample being above the upper limit for hs-CRP; however, it is important to mention that the laboratory ranges are according to the desirable values and not the normal values found in the general population. Therefore, we included them in the analyses by considering that these variations are expected in a relatively healthy population and are useful in the searched associations. In addition, all the values out of range detected in the laboratory tests were double checked (performed twice) in order to verify them. The number and frequency of participants with laboratory values out of range are described in Table 2 and the graphical representation of the outliers of biochemical variables is in Supplementary File S2. All psychological instruments, in addition to the Mini-ECCA and OVIEDO questionnaires, had a Cronbach’s alpha ≥0.60.

In the comparison of sociodemographic variables between sexes, we observed that both sexes were similar in all variables with the exception of daily free hours, which were higher in men than in women, with a median (range) of 4 (1–14) vs. 4 (0–11), *p* = 0.003, as well as the exception of WHR, which was higher in men in comparison with women, with a mean ± SD of 0.84 ± 0.07 vs. 0.77 ± 0.05, *p* < 0.001.

### 3.1. Bivariate Analysis

The bivariate significant correlations in the global sample and/or when separated by sex are presented in Table 3. There we can observe moderate positive correlations between hs-CRP and BMI, WHR, leukocytes, triglycerides, and the liver enzymes GGT and ALP in both sexes. In addition, a moderate negative correlation was found between hs-CRP and HDL. In this bivariate analysis, no significant correlations globally or separated by sex were found for any studied psychological variable.

### 3.2. Multivariate Regression Analysis

The multivariate regression analyses for hs-CRP in the global sample and by sex are presented in Table 4, Table 5 and Table 6.

In the multivariate regression analysis for the global sample, we observed that WHR (b = 0.456) was the most significantly associated variable with hs-CRP; the other positively associated variables in this analysis were the number of monocytes (b = 0.219), alcohol consumption frequency (b = 0.306), uric acid (b = 0.214), anxiety (b = 0.364), emotional regulation (b = 0.231), daily free hours (b = 0.131) and erythrocytes in urine (b = 0.122), while the negatively associated variables found were male sex (b = −0.413), schooling (b = −0.140), positive relations with others (b = −0.172) and depression (b = −0.310). The R of the model was robust: 0.658.

In the case of women, we found that the most significantly associated variable for hs-CRP was the number of neutrophils (b = 0.391), followed by uric acid (b = 0.188), BMI (b = 0.284), erythrocytes in urine (b = 0.251), ALP (b = 0.174), alcohol consumption frequency (b = 0.187), and the presence of leukocyte esterase in urine (b = 0.125). In this case, no psychological variables associated were found. The R of the model was 0.793.

Finally, in the multivariate regression analysis for hs-CRP in men, we observed that the variables positively associated were WHR (b = 0.628), leukocytes in urine (b = 0.279), emotional regulation (b = 0.273), number of monocytes (b = 0.161), anxiety (b = 0.534) and lymphocytes (b = 0.204), while the negatively associated variables with hs-CRP were sleep satisfaction (b = −0.384), schooling (b = −0.244), depression (b = −0.625), with job (b = −0.255) and triglycerides (b = −0.201). The R of the model was 0.806.

## 4. Discussion

The association between psychological and inflammatory markers has been widely studied [3,4,5,6,7,8]; this association has been explained as a bilateral relationship between psychic suffering and inflammation, with some reports showing that inflammation is related to the appearance of depression and suicidal behavior [27,28]. This relationship has also been observed in long-COVID cases, where neuropsychiatric sequelae have been observed in response to hyper and neuroinflammation [29]. On the other hand, it has been shown that psychological stress contributes to systemic chronic inflammation [1,28]; this has been explained by the hyperactivity of the hypothalamic–pituitary–adrenal axis, which leads to low-grade chronic inflammation and mitochondrial disfunction [4,30,31]. In this sense, the negative mood states could contribute to inflammation, which in turn could increase these negative mood states. In addition, a long-term infection or an unhealthy lifestyle related to obesity and sedentarism could tiger inflammation, leading to negative emotional states.

In the present study, we mainly focused on a possible causal relationship between psychological variables and inflammation, with the inclusion of many biochemical confounders, which has been performed in very few studies [15,16], and with a limited number of biochemical variables.

In this study, we observed that HWR and BMI showed a moderate positive correlation with hs-CRP, with a higher correlation with HWR for men and with BMI for women; these findings coincide with most reports that have measured BMI [3,32]. However, the correlation between hs-CRP and these variables (BMI and HWR) separated by sex was not found. Therefore, these results coincide with the reported inflammatory effect of fatty tissue [33], which seems to be mainly distributed in the waist in men and in the entire body in women; this is suggested by the higher values of HWR observed in men than in women in this study (*p* < 0.001), a difference that was not found in BMI between sexes.

These correlations may explain the positive and significant correlations observed between hs-CRP and serum lipids (total cholesterol, triglycerides and LDL) and the two liver enzymes (GGT, ALP), considering that HWR and BMI were positively correlated with these variables; however, elevated levels of liver enzymes have been previously associated with high CPR [34]. The same explanation is proposed to the significant positive correlations between hs-CRP and the sociodemographic variables age, schooling, with children and with job (all these variables showed a positive correlation with BMI in the bivariate analysis). However, it is interesting that some of these correlations change in the multivariate regression analysis, when adjusting for the rest of the variables.

In the bivariate correlations, it is also interesting, although expected, to see the positive correlations between hs-CRP and the total number of leukocytes (being higher in men), particularly with the subpopulations monocytes and neutrophils, which showed positive significant correlations in both sexes. As mentioned, this is an expected correlation when considering that inflammatory processes, including infections, involve an increase in leukocytes and their subpopulations. Another interesting finding is the low but significant positive correlation between hs-CRP and uric acid in both sexes, with it being higher in women, results that coincide with a previous report associating hyperuricemia with hs-CRP in the Mexican population [35]. Finally, we observed a significant correlation between erythrocytes in urine with hs-CRP in women, which is explained by the presence of menstruation and/or urinary infections in participants, which are related with inflammation. No previous reports exploring these associations were found; therefore, it is not possible to us to perform a comparison with the existing literature.

In the bivariate analysis, we did not find any significant correlation between psychological variables and hs-CRP in the global sample or segmented by sex; however, in the multivariate regression analysis, after controlling for confounders, we found that anxiety was positively associated with hs-CRP in global and men’s samples. Likewise, emotional regulation showed a positive and significant association with hs-CRP in these samples. On the other hand, depression was negatively associated with hs-CRP in global and men’s samples, while positive relations with others also showed a negative correlation with hs-CRP in the global sample.

In the case of anxiety, these findings are similar to those reported by Tyefi et al. [6] and Duivis et al. [8], who reported increased levels of hs-CRP in anxiety and depression disorders; in the case of Tayefi et al. [6], it was also reported that this increase was higher in men than in women, which also coincides with our results. In addition, Liukkonen et al. [14] also showed association between hs-CRP and severe anxiety and depression only in men. In the case of depression, we observed that these previous reports used different depression scales than us; in addition, in two of these studies [6,14] they used cut-off values in order to discriminate different levels of depressive symptoms, something that we did not perform considering the small sample size. These methodological differences could be the reason of the opposing correlations found (between depressive symptoms and hs-CPR) between their reports and our report. In addition, we found other studies that used the same depression scale than us (CES-D) [15,36] or a similar scale (PHQ-9) [16], and one of them found no association between clinical depression and the inflammatory marker IL-6 [36] or, similar to our results, two studies showed a negative association between depressive symptoms and hs-CRP [15,16] in multivariate regression analysis, after controlling for confounders. Therefore, the discrepancies observed in the associations reported between depression and hs-CRP may be attributable to the instruments or methodologies employed, being more possible that clinical depression, which usually combines with anxiety problems, is more associated with high hs-CRP, and that depressive symptoms, which are measured in some scales, including the CES-D and PHQ-9, (i.e., “I felt that everything I did was an effort”) is even negatively related with hs-CRP. In addition, we used the short version (10-item) of CES-D, which could be less efficient to detect clinical depressive problems.

On the other hand, it is interesting to note the positive association found between emotional regulation (questions like “I know how to snap out my negative moods”) and hs-CRP in the multivariate regression analysis of the global and men’s samples, which could suggest that people consider themselves able to perform good emotional management of negative emotions because they experience them frequently; in addition, it could be that the repression of negative moods could increase inflammation. However, this association needs to be further explored, considering that no reports that measured this variable were found.

Finally, the negative correlation between positive relations with others and hs-CRP in the global sample is a finding not previously reported that could indicate that emotional support provided by others has an anti-inflammatory effect in both sexes. This is an interesting finding considering that this was the only positive psychological variable negatively associated with hs-CRP, even though positive affect (the positive psychological variable most investigated with inflammatory markers) was also studied but did not reach statistical significance in the multivariate regression analysis.

In the multivariate regression analysis, we also observed that being a woman was positively associated with hs-CRP, which could be explained by the presence of menstruation, which was associated with hs-CRP (by the measurement of erythrocytes in urine), the more frequent presence of asymptomatic urinary infections found in this study, as well as with the presence of more reported diseases in comparison with men reported in previous studies [37]. In addition, the presence of menstruation (detected by the presence of erythrocytes in urine) and urinary infections (detected by the presence of leukocytes and leukocyte esterase in urine) was positively associated with hs-CRP in the global and/or sex-segmented multivariate analyses, which corroborate that these conditions are important contributors of inflammation.

Considering the inclusion of other non-psychological variables in the multivariate regression analysis of the global sample, including the frequency of alcohol consumption, which was positively associated with hs-CRP in the global and women’s samples, is explained by the possible irritant effect that alcohol produces on gastric and/or other organs, leading to inflammation; however, this association was not found in previous studies exploring alcohol consumption and CRP [14,16].

On the other hand, the negative association of the variable schooling with hs-CRP in the global and men’s samples could be explained by a better socioeconomic position throughout life, which is also related with better familiar conditions that would be associated with less adverse events in life and diminished inflammation; in this regard, we found reports that associate adverse life events with inflammation and the presence of chronic diseases [38]. Finally, the positive correlation between daily free hours with hs-CRP in the global sample could be explained by a possible association between daily free hours and negative moods and/or unhealthy lifestyles; this finding coincides with a previous report of our research group, where a low positive correlation was detected between the inflammatory marker erythrocyte sedimentation rate (ESR) and daily free hours in patients with rheumatoid arthritis [39]. However, further studies are necessary in order to confirm these possibilities.

In the case of men, the negative correlations in the multivariate regression analysis between sleep satisfaction and hs-CRP can be explained by considering that sleep promotes a better mood, with less anxiety, and therefore less inflammation; in this case, we did not find studies related exploring this association. Likewise, the negative correlation with having a job could be also related with the better emotional states that an occupation brings, although more studies are needed in order to confirm this explanation. Finally, the negative correlation with triglycerides (opposite to the bivariate correlations) could be a spurious association (considering the small sample size) or indeed could be a causal association that needs to be corroborated with larger sample sizes.

The present results yield much information related with potential clinical applications in preventing programs addressed to diminish inflammation in healthy and/or ill populations; for instance, the inclusion of programs intended to diminish BMI and WHR (mainly through lifestyle modifications), along with the increase in perceived emotional support by improving the positive relations with others, together with psychological trainings for anxiety management, could be useful for this purpose. In addition, the diminishment of inflammation would prevent the appearance of chronic diseases or its progression. Furthermore, the improvement in lifestyle and mental health would also diminish the likelihood of alcohol consumption, with was another related variable with hs-CRP.

The main limitation of the study is the small sample size, which, along with the inclusion of several variables, could increase the appearance of spurious correlations that do not correspond to the real associations among the studied variables; this is due to the random error. However, the strengths of the study are in the inclusion of many confounders (by the strict control in the inclusion criteria, as well as with the measurement of personal, psychological and biochemical variables) and the segmented analysis by sex, which increase the possibility to detect more reliable results and specific for each sex, which should be confirmed with larger sample sizes. The inclusion of a larger sample size would have permitted us to detect potential interactions among variables.

In this sense, although the small sample sizes could increase the random error, the adjusting for many confounders could also diminish the confusing bias. Another limitation is the cross-sectional design which does not permit to detect causal relationships between the measured variables. These causal relationships could be only detected with longitudinal studies.

## 5. Conclusions

In conclusion, we confirmed the moderate positive correlation between hs-CRP and BMI, HWR, uric acid and hepatic enzymes. We additionally detected the positive correlation between leukocytes and their subpopulations (mainly monocytes and neutrophils) with hs-CRP in both sexes. We also detected positive correlations between hs-CRP and the presence of erythrocytes, leukocytes and leukocyte esterase in urine, correlations that have not been previously researched. Finally, we corroborated the association between anxiety symptoms and hs-CRP, in a healthy population, after controlling for many confounders, and showed that some symptoms of depression are negatively correlated with hs-CRP. In addition, the variable “positive relation with others” was the only positive psychological variable negatively associated with hs-CRP in the global sample. All these results lead us to confirm that hs-CRP, a marker of inflammation, is conditioned by many factors, including psychological ones, where anxiety was the most important variable associated with hs-CRP, mainly in men. In addition, positive relations with others is a positive psychological variable that can diminish inflammation in both sexes. However, further longitudinal studies with larger sample sizes that include the same or even more confounding variables will clarify these observations.

## Figures and Tables

**Table 1:** Descriptive data of sociodemographic and psychological variables.

Variable	Global Sample (*n* = 172)	Women (*n* = 91)	Men (*n* = 81)
With romantic partner, *n* (%)	89 (51.7)	46 (50.5)	43 (53.1)
With children, *n* (%)	35 (20.3)	24 (26.4)	11 (13.6)
With job, *n* (%)	89 (51.7)	51 (56.0)	38 (46.9)
Schooling, *n* (%) - Elementary school - Secondary - Preparatory - University (Bachelor’s degree) - Master’s degree - Ph.D. degree	1 (0.6) 5 (2.9) 99 (57.5) 53 (30.8) 13 (7.6) 1 (0.6)	1 (1.1) 3 (3.3) 48 (52.7) 32 (35.2) 7 (7.7) 0 (0.0)	0 (0.0) 2 (2.5) 51 (63.0) 21 (25.9) 6 (7.4) 1 (1.2)
Socioeconomic level, *n* (%) - Very low - Low - Average - High - Very high	2 (1.2) 32 (18.6) 133 (77.3) 5 (2.9) 0 (0.0)	0 (0.0) 17 (18.7) 73 (80.2) 1 (1.1) 0 (0.0)	2 (2.5) 15 (18.5) 60 (74.1) 4 (4.9) 0 (0.0)
Monthly extra money, mean ± SD *	3.06 ± 1.25	2.95 ± 1.17	3.19 ± 1.32
Smoking frequency, median (range) *	0 (0–4)	0 (0–4)	0 (0–4)
Alcohol consumption frequency, mean ± SD *	1.45 ± 0.90	1.46 ± 0.87	1.44 ± 0.94
Daily free hours, median (range) *	4 (0–14)	4 (0–11)	4 (0–14)
Daily physical activity hours, median (range) *	1 (0–5)	1 (0–5)	1 (0–4)
Body mass index (BMI), mean ± SD	24.38 ± 3.96	23.99 ± 3.73	24.82 ± 4.19
Waist to hip ratio (WHR), mean ± SD	0.80 ± 0.07	0.77 ± 0.05	0.84 ± 0.07
Sleep satisfaction (OVIEDO scale), median (range) *	4.03 ± 1.49	3.90 ± 1.45	4.17 ± 1.53
Sleep quality (OVIEDO scale), mean ± SD	3.64 ± 0.95	3.57 ± 0.96	3.73 ± 0.93
Quality of food intake (Mini-ECCA scale), mean ± SD *	7.47 ± 2.53	7.73± 2.65	7.17 ± 2.38
Psychological variables			
Anxiety (GAD-7), mean ± SD *	1.06 ± 0.69	1.19 ± 0.74	0.90 ± 0.59
Depression (CES-D), mean ± SD *	1.88 ± 0.53	1.94 ± 0.56	1.80 ± 0.47
Psychological wellbeing (PWB), mean ± SD - Self-acceptance * - Autonomy * - Purpose in life * - Positive relations with others * - Personal growth * - Environmental mastery	4.71 ± 1.15 4.11 ± 1.01 4.59 ± 1.18 4.81 ± 1.02 5.05 ± 0.94 4.42 ± 1.04	4.63 ± 1.19 3.92 ± 0.97 4.57 ± 1.19 4.89 ± 1±.04 5.12 ± 0.93 4.40 ± 1.08	4.79 ± 1.10 4.32 ± 1.02 4.61 ± 1.17 4.71 ± 0.98 4.98 ± 0.96 4.44 ± 1.01
Emotional intelligence (TIEQUE), mean ± SD - Assertiveness - Emotion regulation - Self-motivation - Emotion perception *	4.87 ± 1.05 4.93 ± 1.21 5.08 ± 1.19 4.98 ± 1.41	4.66 ± 0.96 4.85 ± 1.17 5.19 ± 1.17 4.92 ± 1.46	5.11 ± 1.10 5.03 ± 1.25 4.95 ± 1.21 5.05 ± 1.37
Positive emotions (PSS), mean ± SD	3.73 ± 0.59	3.66 ± 0.64	3.81 ± 0.54
Negative emotions (PSS), mean ± SD *	2.51 ± 0.62	2.60 ± 0.64	2.40 ± 0.59
Optimism (LOT-R), mean ± SD	3.69 ± 0.71	3.68 ± 0.74	3.68 ± 0.68

* Variables with non-parametric distribution. Monthly extra money, with five categories, from nothing to more than 150 dollars, as well as smoking and alcohol consumption frequencies were measured, from 0–4 (never to more than 4 times in the week); also measured were sleep satisfaction (OVIEDO scale), from 1–7 (very unsatisfied to very satisfied), sleep quality (OVIEDO scale), from 1–5 (low quality to high quality), quality of food intake (Mini-ECCA scale) from 1–12 (very low quality to very high quality), anxiety (GAD-7 scale), from 0–3 (never to almost all the days), depression (CES-D scale), from 1–4 (none day to all the days), subscales from psychological wellbeing (PWB), from 1–6 (totally disagree to totally agree), emotional intelligence (TEIQUE scale), from 1–7 (totally disagree to totally agree), positive and negative emotions (PSS scale), from 1–5 (never to almost always), and optimism (LOT-R), from 1–5 (totally disagree to totally agree).

**Table 2:** Descriptive data of the laboratory variables studied.

Variable	Women (*n* = 91)	Men (*n* = 81)	Laboratory Reference Values	Participants Out of Range *n* (%)
hs-CRP (mg/L), median (range) *	0.95 (0.12–11.57)	0.85 (0.14–36.00)	≤3.0	31 (18.0)
Leukocytes (10^3^/μL), mean ± SD - Lymphocytes - Monocytes - Neutrophils - Eosinophils * - Basophils *	6.80 ± 1.48 2.23 ± 0.53 0.49 ± 0.14 3.88 ± 1.18 0.14 ± 0.12 0.04 ± 0.02	6.58 ±1.81 2.17 ± 0.55 0.53 ± 0.16 3.68 ± 1.44 0.13 ± 0.09 0.05 ± 0.02	5.0–10.00 1.0–4.20 0.10–1.00 1.50–7.00 0.05–0.40 0.01–0.05	28 (16.3) 1 (0.6) 1 (0.6) 4 (2.3) 21 (12.2) 42 (24.4)
Hemoglobin (g/dL), mean ± SD	13.83 ± 1.14	16.25 ± 0.99	W: 12.00–16.00 M: 14.00–17.00	7 (7.7) 17 (21.0)
Platelets (10^3^/μL), mean ± SD	276.47 ± 53.05	261.48 ± 52.30	141.00–400.00	3 (1.7)
Glucose (g/dL), mean ± SD	87.33 ± 8.58	89.52 ± 7.86	74.00–106.00	11 (6.4)
Urea (mg/dL), mean ± SD *	25.49 ± 6.57	28.38 ± 7.04	16.6–48.5	6 (3.5)
Blood urea nitrogen (BUN), mg/dL, mean ± SD *	11.91 ± 3.07	13.26 ± 3.29	6.0–20.0	8 (4.7)
Creatinine (mg/dL), mean ± SD	0.74 ± 0.11	0.95 ± 0.13	W: 0.50–0.90 M: 0.70–1.20	8 (8.8) 4 (5.0)
Uric acid (mg/dL) mean ± SD	4.22 ± 0.99	6.02 ± 1-12	W: 2.4–5.7 M: 3.4–7.0	7 (7.7) 14 (17.3)
Lipid levels (mg/dL), mean ± SD - Total cholesterol - High density lipoprotein (HDL) - Low density lipoprotein (LDL) - Triglycerides	165.97 ± 29.26 52.01 ± 11.74 95.40 ± 24.32 92.77 ± 43.73	176.55 ± 36.23 44.63 ± 10.29 108.85 ± 29.53 118.65 ± 68.93	≤200.00 W ≥ 45.00, M ≥ 35.00 ≤100.00 ≤150.00	36 (20.9) 31 (34.1), 13 (16.0) 82 (47.7) 34 (19.8)
Bilirubin (mg/dL), mean ± SD - Total bilirubin * - Direct bilirubin * - Indirect bilirubin *	0.48 ± 0.28 0.20 ± 0.08 0.29 ± 0.20	0.72 ± 0.36 0.26 ± 0.10 0.46 ± 0.27	≤ 1.20 ≤ 0.30 0.10–1.00	9 (5.2) 27 (15.7) 9 (5.2)
Liver enzymes (U/L), mean ± SD - AST * - ALT * - GGT * - ALP - LDH *	20.05 ± 16.08 17.55 ± 15.43 16.16 ± 7.61 78.58 ± 17.59 169.69 ± 31.89	29.70 ± 39.57 28.66± 21.19 27.40 ± 22.47 97.26 ± 26.88 186.31 ±100.56	W ≤ 32.00, M ≤ 40.00 W ≤ 33.00, M ≤ 41.00 W ≤ 40.00, M ≤ 60.00 W ≤ 104.00, M ≤ 129.00 W ≤ 214.00, M ≤ 225.00	4 (4.4), 7 (8.6) 6 (6.5), 11 (13.6) 2 (2.2), 4 (4.9) 5 (5.5), 12 (14.8) 4 (4.4), 5 (6.2)
Urine exam, median (range) - Leukocyte esterase - Nitrites - Erythrocytes *	0 (0–500) 0 (0–1) 0 (0–20)	0 (0–100) 0 (0–0) 0 (0–20)	0 0 0	50 (29.1) 4 (2.3) 67 (39.0)

* Variables with non-parametric distribution. AST: aspartate aminotransferase, ALT: alanine aminotransferase, GGT: gamma glutamyl transferase, ALP: Alkaline phosphatase, LDH: lactate dehydrogenase. W: women, M: men.

**Table 3:** Significant bivariate correlations between studied variables and hs-CRP globally and/or separated by sex.

Variable	Women (*n* = 91)	Men (*n* = 81)	Global Sample (*n* = 172)
Age	0.248 *	0.147	0.222 **
Schooling	0.184	0.134	0.166 *
With children	0.213 *	0.189	0.225 **
With job	0.252 *	0.151	0.217 **
Body mass index (BMI)	0.544 **	0.396 **	0.489 **
Waist/hip ratio (WHR)	0.299 **	0.413 **	0.240 **
Leukocytes	0.343 **	0.439 **	0.375 **
Erythrocytes	0.130	0.213 *	0.063
Platelets	0.226 *	0.230 *	0.237 **
Lymphocytes	0.117	0.348 **	0.217 **
Monocytes	0.245 *	0.291 **	0.250 **
Eosinophils	0.209*	0.163	0.178 *
Neutrophils	0.343 **	0.368 **	0.341 **
Uric acid	0.398 **	0.292 **	0.201 **
Cholesterol	0.239 *	0.141	0.188 *
Triglycerides	0.475 **	0.316 **	0.386 **
High-density lipoprotein (HDL)	−0.351 **	−0.494 **	−0.359 **
Low-density lipoprotein (LDL)	0.295 **	0.173	0.223 **
Gamma glutamyl transferase (GGT)	0.458 **	0.379 **	0.342 **
Alkaline phosphatase (ALP)	0.365 **	0.294 **	0.282 **
lactate dehydrogenase (LDH)	0.080	0.329 **	0.186 *
Erythrocytes in urine	0.256 **	0.201	0.237 **

* *p* value < 0.05, ** *p* value < 0.01. Correlations performed with Spearman correlation test.

**Table 4:** Multivariate regression analysis for hs-CRP in the global sample.

Variable	B	Beta Coefficient	Significance	Tolerance	Change in R^2^
Constant	−17.889	-	0.000	-	-
Waist/hip ratio (WHR)	24.704	0.456	0.000	0.543	0.135
Sex	−3.095	−0.413	0.000	0.454	0.050
Monocytes	5.473	0.219	0.001	0.911	0.051
Alcohol consumption frequency	1.276	0.306	0.000	0.866	0.053
Uric acid	0.576	0.214	0.015	0.482	0.029
Anxiety (GAD-7)	1.974	0.364	0.000	0.363	0.016
Schooling	−0.726	−0.140	0.038	0.824	0.014
Emotional regulation	0.712	0.231	0.001	0.735	0.019
Daily free hours	0.194	0.131	0.045	0.876	0.011
Erythrocytes in urine	0.150	0.122	0.057	0.905	0.011
Positive relations with others	−0.628	−0.172	0.021	0.667	0.013
Depression (CES-D)	−2.191	−0.310	0.003	0.340	0.016

R of the model = 0.658, R^2^ = 0.433. Multiple regression analysis performed with the stepwise method. Sex codification: 1 = women, 2 = men.

**Table 5:** Multivariate regression analysis for hs-CRP in women.

Variable	B	Beta Coefficient	Significance	Tolerance	Change in R^2^
Constant	−10.685	-	0.000	-	-
Neutrophils	0.799	0.391	0.000	0.931	0.265
Uric acid	0.469	0.188	0.013	0.848	0.133
Body mass index (BMI)	0.190	0.284	0.000	0.894	0.078
Erythrocytes in urine	0.177	0.251	0.001	0.884	0.076
Alkaline phosphatase (ALP)	0.024	0.174	0.016	0.927	0.035
Alcohol consumption frequency	0.521	0.187	0.009	0.928	0.028
Leukocyte esterase	0.002	0.125	0.087	0.883	0.014

R of the model: 0.793, R^2^ = 0.629. Multiple regression analysis performed with the stepwise method.

**Table 6:** Multivariate regression analysis for hs-CRP in men.

Variable	B	Beta Coefficient	Significance	Tolerance	Change in R^2^
Constant	−27.014	-	0.000	-	-
Waist/hip ratio (WHR)	43.430	0.628	0.000	0.507	0.248
Leukocytes in urine	1.262	0.279	0.001	0.819	0.045
Emotional regulation	1.048	0.273	0.001	0.882	0.035
Sleep satisfaction	−1.220	−0.384	0.000	0.654	0.033
Schooling	−1.667	−0.244	0.006	0.703	0.045
Monocytes	4.826	0.161	0.047	0.824	0.023
Depression (CES-D)	−6.330	−0.625	0.000	0.349	0.027
Anxiety (GAD-7)	4.329	0.534	0.000	0.437	0.078
With job	−2.460	−0.255	0.005	0.693	0.027
Lymphocytes	1.795	0.204	0.022	0.696	0.019
Triglycerides	−0.014	−0.201	0.041	0.563	0.023

R of the model = 0.806, R^2^ = 0.650. Multiple regression analysis performed with the stepwise method.

## Data Availability

Not applicable.

## Supplementary Materials

The following supporting information can be downloaded at: https://www.mdpi.com/article/10.3390/jpm13050876/s1.
